# Beyond FMLA: Making the Case for State-Level Paid Family Leave, the Path to Policy Adoption

**DOI:** 10.1093/geroni/igaa057.2553

**Published:** 2020-12-16

**Authors:** Eugenie Stephenson

**Affiliations:** University of Maryland, Baltimore County, Baltimore, Maryland, United States

## Abstract

The Family Medical Leave Act (FMLA) was established in 1993, and while limited in scope, it is the only national policy in place to support individuals that need to take time away from work to care for a sick family member. Paid Family Leave, a state-level mechanism of FMLA, creates a safety net to support these workers. Despite its potential impact, such programs currently exist in only 6 states and the District of Columbia. Further, even within these states, individuals are often unaware of the policy's existence. A systematic review of peer reviewed literature from 1994-2019 explores the predictors of the adoption and implementation of state-level Paid Family Leave and public awareness of Paid Family Leave programs in these states. This presentation will explore these findings and their implications on future state level adoption, implementation and awareness of Paid Family Leave.

